# Supplementary material for: Nonclonal Emergence of Colistin Resistance Associated with Mutations in the BasRS Two-Component System in Escherichia coli Bloodstream Isolates
Source: mSphere. 2020 Mar 11;5(2):e00143-20. doi: 10.1128/mSphere.00143-20 (PMC7067592; doi:10.1128/mSphere.00143-20)
Supplement: TABLE S1 [file mSphere.00143-20-st001.docx]

| **Strain** | **MLST** | **GenBank assembly accession number** | **Organism** | **Closest match** |
| --- | --- | --- | --- | --- |
| 42 | 414 | GCA_000027125.1 | *Escherichia coli* |  |
| 536 | 127 | GCA_000013305.1 | *Escherichia coli* |  |
| 789 | 88 | GCA_000819645.1 | *Escherichia coli* |  |
| 1303 | 10 | GCA_000829985.1 | *Escherichia coli* |  |
| 6409 | 10 | GCA_000814145.2 | *Escherichia coli* | E3090 |
| 11128 | 16 | GCA_000010765.1 | *Escherichia coli* |  |
| 11368 | 21 | GCA_000091005.1 | *Escherichia coli* |  |
| 12009 | 17 | GCA_000010745.1 | *Escherichia coli* |  |
| 55989 | 3762 | GCA_000026245.1 | *Escherichia coli* |  |
| 180‑PT54 | 5638 | GCA_001650275.1 | *Escherichia coli* |  |
| 2009C‑3133 | 117 | GCA_001420955.1 | *Escherichia coli* |  |
| 2009EL‑2050 | 678 | GCA_000299255.1 | *Escherichia coli* |  |
| 2009EL‑2071 | 678 | GCA_000299475.1 | *Escherichia coli* |  |
| 2011C‑3493 | 678 | GCA_000299455.1 | *Escherichia coli* |  |
| 2011C‑3911 | 1727 | GCA_001644725.1 | *Escherichia coli* |  |
| 2012C‑4227 | 119 | GCA_001420935.1 | *Escherichia coli* |  |
| 2013C‑4465 | 335 | GCA_001644745.1 | *Escherichia coli* |  |
| 28RC1 | 11 | GCA_001612475.1 | *Escherichia coli* |  |
| 644‑PT8 | N.D. | GCA_001650295.1 | *Escherichia coli* |  |
| 94‑3024 | 672 | GCA_000801185.2 | *Escherichia coli* |  |
| ABU 83972 | 73 | GCA_000148365.1 | *Escherichia coli* |  |
| ACN001 | 23 | GCA_001051135.1 | *Escherichia coli* |  |
| ACN002 | 23 | GCA_001515725.1 | *Escherichia coli* |  |
| APEC IMT5155 | N.D. | GCA_000813165.1 | *Escherichia coli* |  |
| APEC O1 | 95 | GCA_000014845.1 | *Escherichia coli* |  |
| APEC O78 | 23 | GCA_000332755.1 | *Escherichia coli* |  |
| ATCC 25922 | 73 | GCA_000743255.1 | *Escherichia coli* | F2745 |
| ATCC 8739 | 3021 | GCA_000019385.1 | *Escherichia coli* |  |
| B7A | N.D. | GCA_000725265.1 | *Escherichia coli* |  |
| BL21 (TaKaRa) | 93 | GCA_000833145.1 | *Escherichia coli* |  |
| BL21(DE3) | 93 | GCA_000009565.2 | *Escherichia coli* |  |
| BL21(DE3) | 93 | GCA_000022665.2 | *Escherichia coli* |  |
| BL21‑Gold(DE3)pLysS AG | 93 | GCA_000023665.1 | *Escherichia coli* |  |
| C227‑11 | 678 | GCA_000986765.1 | *Escherichia coli* |  |
| C2566 | 93 | GCA_001559615.1 | *Escherichia coli* |  |
| C3026 | 10 | GCA_001559675.1 | *Escherichia coli* |  |
| C3029 | 93 | GCA_001559635.1 | *Escherichia coli* |  |
| C321.deltaA | 10 | GCA_000474035.1 | *Escherichia coli* |  |
| C41(DE3) | 93 | GCA_000830035.1 | *Escherichia coli* |  |
| C43(DE3) | 93 | GCA_001039415.1 | *Escherichia coli* |  |
| CB9615 | 335 | GCA_000025165.1 | *Escherichia coli* |  |
| CD306 | 131 | GCA_001513615.1 | *Escherichia coli* |  |
| CE10 | 62 | GCA_000227625.1 | *Escherichia coli* |  |
| CFSAN029787 | 99 | GCA_001007915.1 | *Escherichia coli* |  |
| CFT073 | 73 | GCA_000007445.1 | *Escherichia coli* |  |
| CI5 | 5082 | GCA_000971615.1 | *Escherichia coli* |  |
| clone D i14 | 73 | GCA_000233895.1 | *Escherichia coli* |  |
| clone D i2 | 73 | GCA_000233875.1 | *Escherichia coli* |  |
| CQSW20 | 1060 | GCA_001455385.1 | *Escherichia coli* |  |
| DH1 #1 | 1060 | GCA_000023365.1 | *Escherichia coli* |  |
| DH1 #2 | 1060 | GCA_000270105.1 | *Escherichia coli* |  |
| DH1Ec095 | 1060 | GCA_001183645.1 | *Escherichia coli* |  |
| DH1Ec104 | 1060 | GCA_001183665.1 | *Escherichia coli* |  |
| DH1Ec169 | 1060 | GCA_001183685.1 | *Escherichia coli* |  |
| DHB4 | 10 | GCA_001559655.1 | *Escherichia coli* |  |
| E2348/69 | 15 | GCA_000026545.1 | *Escherichia coli* |  |
| E24377A | 1132 | GCA_000017745.1 | *Escherichia coli* |  |
| EC4115 | 11 | GCA_000021125.1 | *Escherichia coli* |  |
| EC958 | 131 | GCA_000285655.3 | *Escherichia coli* | **G821/H2129** |
| ECC‑1470 | 847 | GCA_000831565.1 | *Escherichia coli* |  |
| Eco889 | 131 | GCA_001663475.1 | *Escherichia coli* |  |
| Ecol_448 | 131 | GCA_001618365.1 | *Escherichia coli* |  |
| Ecol_732 | 131 | GCA_001617565.1 | *Escherichia coli* | **I1121** |
| Ecol_743 | 131 | GCA_001618325.1 | *Escherichia coli* |  |
| Ecol_745 | 131 | GCA_001618345.1 | *Escherichia coli* |  |
| ECONIH1 | 648 | GCA_000784925.1 | *Escherichia coli* |  |
| EDL933 | 11 | GCA_000732965.1 | *Escherichia coli* |  |
| ER1821R | 10 | GCA_001663075.1 | *Escherichia coli* |  |
| ER2796 | 10 | GCA_000800215.1 | *Escherichia coli* |  |
| ER3413 | 10 | GCA_000800765.1 | *Escherichia coli* |  |
| ER3435 | N.D. | GCA_000974885.1 | *Escherichia coli* |  |
| ER3440 | N.D. | GCA_000974465.1 | *Escherichia coli* |  |
| ER3445 | N.D. | GCA_000974535.1 | *Escherichia coli* |  |
| ER3446 | N.D. | GCA_000974825.1 | *Escherichia coli* |  |
| ER3454 | N.D. | GCA_000974405.1 | *Escherichia coli* |  |
| ER3466 | N.D. | GCA_000974575.1 | *Escherichia coli* |  |
| ER3475 | N.D. | GCA_000974865.1 | *Escherichia coli* |  |
| ER3476 | N.D. | GCA_000974505.1 | *Escherichia coli* |  |
| ETEC H10407 | 48 | GCA_000210475.1 | *Escherichia coli* |  |
| FRIK2069 | 11 | GCA_001651925.1 | *Escherichia coli* |  |
| FRIK2455 | 11 | GCA_001651965.1 | *Escherichia coli* |  |
| FRIK2533 | 11 | GCA_001651945.1 | *Escherichia coli* |  |
| G749 | 131 | GCA_001566635.1 | *Escherichia coli* |  |
| HS | 46 | GCA_000017765.1 | *Escherichia coli* |  |
| HUSEC2011 | 678 | GCA_000967155.1 | *Escherichia coli* |  |
| IAI1 | 1128 | GCA_000026265.1 | *Escherichia coli* | E650 |
| IAI39 | 62 | GCA_000026345.1 | *Escherichia coli* |  |
| IHE3034 | 95 | GCA_000025745.1 | *Escherichia coli* |  |
| JEONG‑1266 | 11 | GCA_001558995.2 | *Escherichia coli* |  |
| JJ1886 | 131 | GCA_000493755.1 | *Escherichia coli* |  |
| JJ1887 | 131 | GCA_001593565.1 | *Escherichia coli* |  |
| JJ1897 | 131 | GCA_001513655.1 | *Escherichia coli* |  |
| JJ2434 | 131 | GCA_001513635.1 | *Escherichia coli* |  |
| JW5437‑1 substr. MG1655 | 10 | GCA_001566335.1 | *Escherichia coli* |  |
| AG100 | 7415 | GCA_000981485.1 | *Escherichia coli* |  |
| BW25113 | 1996 | GCA_000750555.1 | *Escherichia coli* |  |
| BW2952 | 5967 | GCA_000022345.1 | *Escherichia coli* |  |
| DH10B | 4638 | GCA_000019425.1 | *Escherichia coli* |  |
| GM4792 #1 | 10 | GCA_001020945.2 | *Escherichia coli* |  |
| GM4792 #2 | 10 | GCA_001021005.2 | *Escherichia coli* |  |
| HMS174 | 10 | GCA_000953515.1 | *Escherichia coli* |  |
| MC4100 | 10 | GCA_000499485.1 | *Escherichia coli* |  |
| MDS42 | 1060 | GCA_000350185.1 | *Escherichia coli* |  |
| MG1655 #1 | 10 | GCA_000005845.2 | *Escherichia coli* |  |
| MG1655 #2 | 10 | GCA_000801205.1 | *Escherichia coli* |  |
| MG1655 #3 | 1060 | GCA_001308065.1 | *Escherichia coli* |  |
| MG1655 #4 | 10 | GCA_001544635.1 | *Escherichia coli* |  |
| MG1655_TMP32XR1 | 10 | GCA_001308125.1 | *Escherichia coli* |  |
| MG1655_TMP32XR2 | 10 | GCA_001308165.1 | *Escherichia coli* |  |
| RV308 | 10 | GCA_000952955.1 | *Escherichia coli* |  |
| W3110 | 10 | GCA_000010245.1 | *Escherichia coli* |  |
| KLY | 10 | GCA_000725305.1 | *Escherichia coli* |  |
| KO11 | 1079 | GCA_000147855.3 | *Escherichia coli* |  |
| KO11FL | 1079 | GCA_000258025.1 | *Escherichia coli* |  |
| LF82 | 135 | GCA_000284495.1 | *Escherichia coli* |  |
| LY180 | 1079 | GCA_000468515.1 | *Escherichia coli* |  |
| MNCRE44 | 131 | GCA_000931565.1 | *Escherichia coli* |  |
| MRE600 | N.D. | GCA_001542675.2 | *Escherichia coli* |  |
| MVAST0167 | 131 | GCA_001566655.1 | *Escherichia coli* |  |
| NA114 | 131 | GCA_000214765.2 | *Escherichia coli* |  |
| NCM3722 | 10 | GCA_001043215.1 | *Escherichia coli* |  |
| NGF1 | 998 | GCA_001660585.1 | *Escherichia coli* |  |
| Nissle 1917 | 73 | GCA_000714595.1 | *Escherichia coli* |  |
| NRG 857C | 135 | GCA_000183345.1 | *Escherichia coli* |  |
| P12b | 10 | GCA_000257275.1 | *Escherichia coli* |  |
| PCN033 | 5147 | GCA_000219515.3 | *Escherichia coli* | D2373 |
| PCN061 | 46 | GCA_001029125.1 | *Escherichia coli* |  |
| REL606 | 93 | GCA_000017985.1 | *Escherichia coli* |  |
| RM12579 | 335 | GCA_000245515.1 | *Escherichia coli* |  |
| RM12581 | 32 | GCA_000671295.1 | *Escherichia coli* |  |
| RM12761 | 6130 | GCA_000662395.1 | *Escherichia coli* |  |
| RM13514 | 32 | GCA_000520035.1 | *Escherichia coli* |  |
| RM13516 | 6130 | GCA_000520055.1 | *Escherichia coli* |  |
| RM9387 | 2773 | GCA_000801165.1 | *Escherichia coli* |  |
| RR1 | 10 | GCA_001276585.1 | *Escherichia coli* |  |
| RS218 | N.D. | GCA_000800845.2 | *Escherichia coli* |  |
| S51 | 7060 | GCA_001660565.1 | *Escherichia coli* |  |
| S88 | 95 | GCA_000026285.1 | *Escherichia coli* |  |
| Sakai | 11 | GCA_000008865.1 | *Escherichia coli* |  |
| Sanji | 167 | GCA_001610755.1 | *Escherichia coli* | **Z821** |
| Santai | 1011 | GCA_000827105.1 | *Escherichia coli* |  |
| SaT040 | 131 | GCA_001566615.1 | *Escherichia coli* |  |
| SE11 | 156 | GCA_000010385.1 | *Escherichia coli* |  |
| SE15 | 131 | GCA_000010485.1 | *Escherichia coli* |  |
| SEC470 | 48 | GCA_000987875.1 | *Escherichia coli* |  |
| SF‑088 | 95 | GCA_001280325.1 | *Escherichia coli* |  |
| SF‑166 | 95 | GCA_001280385.1 | *Escherichia coli* |  |
| SF‑173 | 95 | GCA_001280405.1 | *Escherichia coli* |  |
| SF‑468 | 95 | GCA_001280345.1 | *Escherichia coli* |  |
| SMS‑3‑5 | 354 | GCA_000019645.1 | *Escherichia coli* | E2372 |
| SQ110 | 10 | GCA_000988425.1 | *Escherichia coli* |  |
| SQ171 | 10 | GCA_000988445.1 | *Escherichia coli* |  |
| SQ2203 | 10 | GCA_000988465.1 | *Escherichia coli* |  |
| SQ37 | 10 | GCA_000988355.1 | *Escherichia coli* |  |
| SQ88 | 10 | GCA_000988385.1 | *Escherichia coli* |  |
| SRCC 1675 | 11 | GCA_001612495.1 | *Escherichia coli* |  |
| SS17 | 11 | GCA_000730345.1 | *Escherichia coli* |  |
| SS52 | 11 | GCA_000803705.1 | *Escherichia coli* |  |
| ST2747 | 6131 | GCA_000599665.1 | *Escherichia coli* |  |
| ST2747 | 6131 | GCA_000599685.1 | *Escherichia coli* |  |
| ST2747 | 6131 | GCA_000599705.1 | *Escherichia coli* |  |
| ST540 #1 | 540 | GCA_000597845.1 | *Escherichia coli* |  |
| ST540 #2 | 540 | GCA_000599625.1 | *Escherichia coli* |  |
| ST540 #3 | 540 | GCA_000599645.1 | *Escherichia coli* |  |
| ST648 | 648 | GCA_001485455.1 | *Escherichia coli* |  |
| TW14359 | 11 | GCA_000022225.1 | *Escherichia coli* |  |
| uk_P46212 | 131 | GCA_001469815.1 | *Escherichia coli* |  |
| UM146 | 643 | GCA_000148605.1 | *Escherichia coli* |  |
| UMNK88 | 100 | GCA_000212715.2 | *Escherichia coli* |  |
| UTI89 | 95 | GCA_000013265.1 | *Escherichia coli* |  |
| VR50 | 10 | GCA_000968515.1 | *Escherichia coli* |  |
| W #1 | 1079 | GCA_000184185.1 | *Escherichia coli* |  |
| W #2 | 1079 | GCA_000258145.1 | *Escherichia coli* |  |
| WS4202 | 11 | GCA_001307215.1 | *Escherichia coli* |  |
| Xuzhou21 | 11 | GCA_000262125.1 | *Escherichia coli* |  |
| YD786 | 410 | GCA_001442495.1 | *Escherichia coli* |  |
| ZH063 | 131 | GCA_001577325.1 | *Escherichia coli* |  |
| ZH193 | 131 | GCA_001566675.1 | *Escherichia coli* |  |
| 24 | 4633 | GCA_001514575.1 | *Escherichia albertii* |  |
| jun‑51 | 678 | GCA_001514595.1 | *Escherichia albertii* |  |
| 94389 | 11 | GCA_001514625.1 | *Escherichia albertii* |  |
| 20H38 | 6057 | GCA_001514555.1 | *Escherichia albertii* |  |
| CB10113 | 6054 | GCA_001514825.1 | *Escherichia albertii* |  |
| CB9791 | 6049 | GCA_001514845.1 | *Escherichia albertii* |  |
| E2675 | 2683 | GCA_001514865.1 | *Escherichia albertii* |  |
| EC03‑127 | 6052 | GCA_001514885.1 | *Escherichia albertii* |  |
| EC03‑195 | 4947 | GCA_001514905.1 | *Escherichia albertii* |  |
| EC05‑160 | 3762 | GCA_001514925.1 | *Escherichia albertii* |  |
| EC05‑44 | 6058 | GCA_001514945.1 | *Escherichia albertii* |  |
| EC05‑81 | 6055 | GCA_001514965.1 | *Escherichia albertii* |  |
| EC06‑170 | N.D. | GCA_001549955.1 | *Escherichia albertii* |  |
| HIPH08472 | N.D. | GCA_001514985.1 | *Escherichia albertii* |  |
| K7394 | 10 | GCA_001515005.1 | *Escherichia albertii* |  |
| K7744 | 10 | GCA_001515025.1 | *Escherichia albertii* |  |
| K7756 | 10 | GCA_001515045.1 | *Escherichia albertii* | A2361 |
| KF1 | 10 | GCA_000512125.1 | *Escherichia albertii* |  |
| KU20110014 | 3762 | GCA_001515065.1 | *Escherichia albertii* |  |
| LMG20976 | 383 | GCA_000759775.1 | *Escherichia albertii* |  |
| NIAH_Bird_13 | 3762 | GCA_001514645.1 | *Escherichia albertii* |  |
| NIAH_Bird_16 | 6056 | GCA_001514665.1 | *Escherichia albertii* |  |
| NIAH_Bird_2 | 4606 | GCA_001514685.1 | *Escherichia albertii* |  |
| NIAH_Bird_23 | 6059 | GCA_001514705.1 | *Escherichia albertii* |  |
| NIAH_Bird_24 | 4634 | GCA_001514725.1 | *Escherichia albertii* |  |
| NIAH_Bird_25 | 5967 | GCA_001514745.1 | *Escherichia albertii* |  |
| NIAH_Bird_26 | N.D. | GCA_001514765.1 | *Escherichia albertii* |  |
| NIAH_Bird_5 | 4736 | GCA_001514785.1 | *Escherichia albertii* |  |
| NIAH_Bird_8 | 2700 | GCA_001514805.1 | *Escherichia albertii* |  |
| TW07627 | 383 | GCA_000155105.1 | *Escherichia albertii* |  |
| TW08933 | 1763 | GCA_000208425.2 | *Escherichia albertii* |  |
| TW15818 | N.D. | GCA_000208505.2 | *Escherichia albertii* |  |
