# Supplementary material for: Nonclonal Emergence of Colistin Resistance Associated with Mutations in the BasRS Two-Component System in Escherichia coli Bloodstream Isolates
Source: mSphere. 2020 Mar 11;5(2):e00143-20. doi: 10.1128/mSphere.00143-20 (PMC7067592; doi:10.1128/mSphere.00143-20)
Supplement: TABLE S2 [file mSphere.00143-20-st002.docx]

| **Primer** | **Sequence (5’‑3’)** | **Reference** |
| --- | --- | --- |
| BW25113 *glmS* Up | ATA TTC AGT CAA TTA CAA ACA TTA | This study |
| BW25113 *glmS* Down | CGA TCT TCT ACA CCG TTC | This study |
| Tn7R | CAC AGC ATA ACT GGA CTG ATT TC | (S1) |
| Tn7R inward | GAA ATC AGT CCA GTT ATG CTG TG | This study |
| Tn7L | ATT AGC TTA CGA CGC TAC ACC C | (S1) |
| Tn7L inward | GGG TGT AGC GTC GTA AGC TAA T | This study |
| BasRS sequencing Fwd | AAA GCC CGT ATC CGC AC | This study |
| BasRS sequencing Rev | GAT CTC ACG CAT GAT GTG GC | This study |
| BasRS deletion BW27848 check Fwd | CAA ACG CAA CAC TAT TCA CAA GAC | This study |
| BasRS deletion BW27848 check Rev | ATC TCT GAC GCG CAT ACT CTC | This study |
| pGRG36 check with Tn7L | ATA TGC ACA GAT GAA AAC GGT G | This study |
| BasRS promoter Fwd | CTT CCT CTA CTG CAT CTG GG | This study |
| BasRS promoter E650 Fwd | CTT CCT CTA CTG CAT TTG GG | This study |
| BasRS promoter A2361 Fwd | TTT TCT CTA CTG CAT CTG GG | This study |
| BasRS promoter Rev | CAC GGT GTT TCC ATC GA | This study |
| BasRS promoter A2361 Rev | CAC GGT ATT TCC ATC AA | This study |
| BasRS genes Fwd | ATG AAA ATT CTG ATT GTT GAA GA | This study |
| BasRS genes Rev | GTT CAG CGT GCT GGT GGT | This study |
| BasRS genes A2361 Rev | ATT CAG CGT GCT GGT CGT | This study |
| BasRS genes overlap Fwd | TGC GCA CTT TGT TCG ATG GAA ACA CCG TGA TGA AAA TTC TGA TTG TTG AAG ACG AT | This study |
| BasRS genes overlap A2361 Fwd | TGC GCA CTT TGT TTG ATG GAA ATA CCG TGA TGA AAA TTC TGA TTG TTG AAG ACG AT | This study |
| BasRS promoter NotI Fwd | TAT CCT GCG GCC GCC TTC CTC TAC TGC ATC TGG G | This study |
| BasRS promoter NotI E650 Fwd | TAT CCT GCG GCC GCC TTC CTC TAC TGC ATT TGG G | This study |
| BasRS promoter NotI A2361 Fwd | TAT CCT GCG GCC GCT TTT CTC TAC TGC ATC TGG G | This study |
| BasRS genes XhoI Rev | TAT CCC CTC GAG GTT CAG CGT GCT GGT GGT | This study |
| BasRS genes XhoI A2361 Rev | TAT CCT CTC GAG ATT CAG CGT GCT GGT CGT | This study |
| BasRS promotor NotI short Fwd | TAT CCT GCG GCC GCC | This study |
| BasRS promotor NotI short A2361 Fwd | TAT CCT GCG GCC GCT | This study |
| BasRS promotor XhoI short Rev | TAT CCC CTC GAG GTT CA | This study |
| BasRS promotor XhoI short A2361 Rev | TAT CCT CTC GAG GTT CA | This study |
| I1121 BasS R10L mutagenesis Fwd | CGA CCA ATA TCG CTG CGC CAA CGG CTG | This study |
| I1121 BasS R10L mutagenesis Rev | GCG CAG AAA ACG CAT CAG ATT CAA TTA G | This study |
| H2129 BasR S53G mutagenesis Fwd | AGC CTG GTG GTA CTG GAT TTA GGC TTA CCC GAT G | This study |
| H2129 BasR S53G mutagenesis Rev | GTA ATG ACC GGC TTC AAG GCT TTG TTC CGC | This study |
| G821 BasS duplication mutagenesis Fwd | TTT CAT TAT CGA GCG TGC TGG | This study |
| G821 BasS duplication mutagenesis Rev | GGT TGT TTA CCG CTG ACG TC | This study |
| G821 BasS duplication check Fwd | ATC TGC TAT CAG GCG GTA CG | This study |
| G821 BasS duplication check Rev | GTT CGT CAT ACG AGG GGA GA | This study |
| Z821 BasS P159A mutagenesis Fwd | GAA CGC CAC TGG CGG GGG TGC GT | This study |
| Z821 BasS P159A mutagenesis Rev | GCA GTT CGT GCG CGA CGT CAG CGG TAA ACA A | This study |

**Reference**

S1. Choi K-H, Schweizer HP. 2006. mini-Tn7 insertion in bacteria with single *att*Tn7 sites: example *Pseudomonas aeruginosa*. Nat Protoc 1:153–161.
